# Supplementary material for: Viral diversity is an obligate consideration in CRISPR/Cas9 designs for targeting the HIV reservoir
Source: BMC Biol. 2018 Jul 11;16:75. doi: 10.1186/s12915-018-0544-1 (PMC6040082; doi:10.1186/s12915-018-0544-1)

**LTR reporter design**

**Amino acid changes**

ATG to GTG, M to V

TGA to GGA, Stop to G

TAG to GAG, Stop to E

TAA to GAA, Stop to E

Underlined = internal start or stop codon

Red = added or altered nucleotides

Green = non-coding Kozak sequence

Blue = sgRNAs (Not comprehensive for all consensus LTRs)

**Group M**

GCCGCCACCATGGAAGGGTTAATTTACTCCAAGAAAAGACAAGAGATCCTTGATCTGTGGGTCTATCACACACAAGGCTACTTCCCTGATTGGCAAAACTACACACCAGGGCCAGGGATCAGATATCCACTGACCTTTGGATGGTGCTTCAAGCTAGTACCAGTTGACCCAGAGGAAGTAGAAGAGGCCAATGAAGGAGAGAACAACTGCTTGTTACACCCT**ATG**AGCCAGCATGGA**ATG**GAGGATGAAGAGAGAGAAGTGTTA**ATG**TGGAAGTTTGACAGCCGCCTAGCACTCAGACAC**ATG**GCCCGAGAGCTGCATCCGGAGTACTACAAAGACTGC**TGA**CACAGAAGTTTCTACAGGACTTTCCGCTGGGGACTTTCCAGGGGGGTGTGGTCTGGGCGGGACTGGGGAGTGGCCAACCCTCAG**ATG**CTGCATATAAGCAGCTGCTTTTCGCCTGTACTGGGTCTCTCTAGT**TAG**ACCAGATCTGAGCCTGGGAGCTCTCTGGCTAGC**TAG**GGAACCCACTGCTTAAGCCTCAATAAAGCTTGCCTTGAGTGCTTTAAG**TAG**TGTGTGCCCGTCTGTTGTGTGACTCTGGTAACTAGAGATCCCTCAGACCATTTTAGTCAGTGTGGAAAATCTCTAGCA


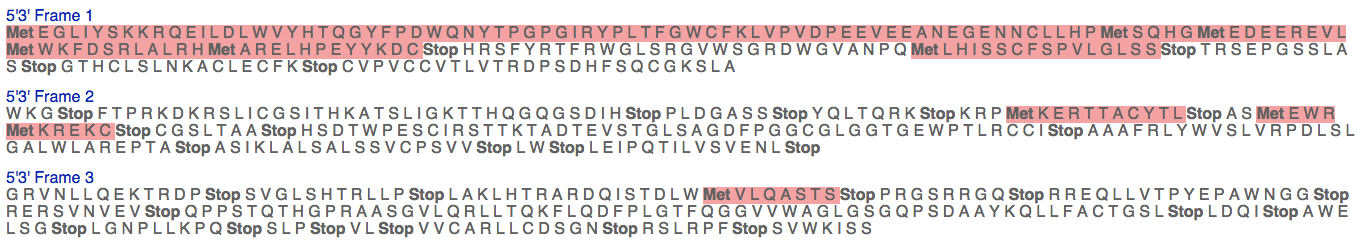


**Group M altered**

GCCGCCACCATGGAAGGGTTAATTTACTCCAAGAAAAGACAAGAGATCCTTGATCTGTGGGTCTATCACACACAAGGCTACTTCCCTGATTGGCAAAACTACACACCAGGGCCAGGGATCAGATATCCACTGACCTTTGGATGGTGCTTCAAGCTAGTACCAGTTGACCCAGAGGAAGTAGAAGAGGCCAATGAAGGAGAGAACAACTGCTTGTTACACCCT**GTG**AGCCAGCATGGA**GTG**GAGGATGAAGAGAGAGAAGTGTTA**GTG**TGGAAGTTTGACAGCCGCCTAGCACTCAGACAC**GTG**GCCCGAGAGCTGCATCCGGAGTACTACAAAGACTGC**GGA**CACAGAAGTTTCTACAGGACTTTCCGCTGGGGACTTTCCAGGGGGGTGTGGTCTGGGCGGGACTGGGGAGTGGCCAACCCTCAG**GTG**CTGCATATAAGCAGCTGCTTTTCGCCTGTACTGGGTCTCTCTAGT**GAG**ACCAGATCTGAGCCTGGGAGCTCTCTGGCTAGC**GAG**GGAACCCACTGCTTAAGCCTCAATAAAGCTTGCCTTGAGTGCTTTAAG**GAG**TGTGTGCCCGTCTGTTGTGTGACTCTGGTAACTAGAGATCCCTCAGACCATTTTAGTCAGTGTGGAAAATCTCTAGCA


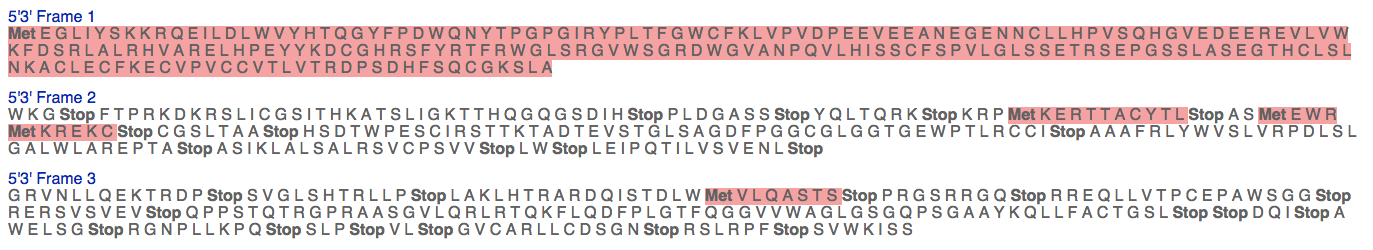


**Amino acid changes**

ATG to GTG, M to V

TGA to GGA, Stop to G

TAG to GAG, Stop to E

TAA to GAA, Stop to E

Underlined = internal start or stop codon

Red = added or altered nucleotides

Green = non-coding Kozak sequence

**SubA**

GCCGCCACCATGGATGGGTTAATTTACTCCAGGAAAAGACAAGAAATCCTTGATCTGTGGGTCTACCACACACAAGGCTACTTCCCTGATTGGCAGAATTACACACCAGGGCCAGGGATCAGATACCCACTAACATTTGGATGGTGCTTCAAGCTAGTACCAGTTGATCCAGATGAAGTAGAGAAGGCTACTGAGGGAGAGAACAACAGCCTATTACACCCTATATGCCAACATGGA**ATG**GATGATGAGGAGAGAGAAACATTA**ATG**TGGAAGTTTGACAGCCGCCTGGCACTAAAACACAGAGCCCGAGAGCTGCATCCGGAGTTCTACAAAGACTGC**TGA**CACAGAAGTTGC**TGA**CAGGGACTTTCCGCTGGGGACTTTCCAGGGGAGGTGTGGTTTGGGCGGAGTTGGGGAGTGGCTAACCCTCAG**ATG**CTGCATATAAGCAGCTGCTTTTCGCCTGTACTGGGTCTCTCTTGT**TAG**ACCAGATCGAGCCTGGGAGCTCTCTGGCTAGCTAGGGAACCCACTGCT**TAA**GCCTCAATAAAGCTTGCCTTGAGTGCTTCAAGTAGTGTGTGCCCGTCTGTTGTG**TGA**CTCTGG**TAA**CTAGAGATCCCTCAGACCACTCTAGACTGTGTAAAAATCTCTAGCAGTGGC


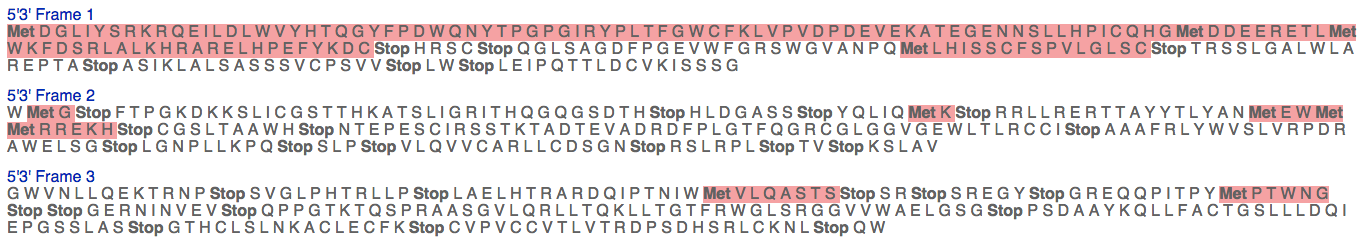


**SubA altered**

GCCGCCACCATGGATGGGTTAATTTACTCCAGGAAAAGACAAGAAATCCTTGATCTGTGGGTCTACCACACACAAGGCTACTTCCCTGATTGGCAGAATTACACACCAGGGCCAGGGATCAGATACCCACTAACATTTGGATGGTGCTTCAAGCTAGTACCAGTTGATCCAGATGAAGTAGAGAAGGCTACTGAGGGAGAGAACAACAGCCTATTACACCCTATATGCCAACATGGA**GTG**GATGATGAGGAGAGAGAAACATTA**GTG**TGGAAGTTTGACAGCCGCCTGGCACTAAAACACAGAGCCCGAGAGCTGCATCCGGAGTTCTACAAAGACTGC**GGA**CACAGAAGTTGC**GGA**CAGGGACTTTCCGCTGGGGACTTTCCAGGGGAGGTGTGGTTTGGGCGGAGTTGGGGAGTGGCTAACCCTCAG**GTG**CTGCATATAAGCAGCTGCTTTTCGCCTGTACTGGGTCTCTCTTGT**GAG**ACCAGATCGAGCCTGGGAGCTCTCTGGCTAGCTAGGGAACCCACTGCT**GAA**GCCTCAATAAAGCTTGCCTTGAGTGCTTCAAGTAGTGTGTGCCCGTCTGTTGTG**GGA**CTCTGG**GAA**CTAGAGATCCCTCAGACCACTCTAGACTGTGTAAAAATCTCTAGCAGTGGC

**
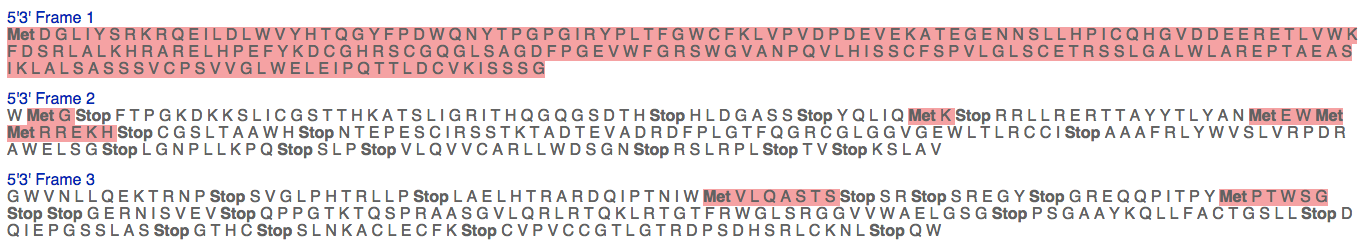
**

**Amino acid changes**

ATG to GTG, M to V

TGA to GGA, Stop to G

TAG to GAG, Stop to E

TAA to GAA, Stop to E

Underlined = internal start or stop codon

Red = added or altered nucleotides

Green = non-coding Kozak sequence

**SubB**

GCCGCCACCATGGAAGGGCTAATTTACTCCCAAAAAAGACAAGATATCCTTGATCTGTGGGTCTACCACACACAAGGCTACTTCCCTGATTGGCAGAACTACACACCAGGGCCAGGGATCAGATATCCACTGACCTTTGGATGGTGCTTCAAGCTAGTACCAGTTGAGCCAGAGAAGGTAGAAGAGGCCAATGAAGGAGAGAACAACAGCTTGTTACACCCT**ATG**AGCCTGCATGGG**ATG**GAGGACCCGGAGAAAGAAGTGTTAGTGTGGAAGTTTGACAGCCGCCTAGCATTTCATCAC**ATG**GCCCGAGAGCTGCATCCGGAGTACTACAAGGACTGC**TGA**CATCGAGCTTTCTACAAGGGACTTTCCGCTGGGGACTTTCCAGGGAGGCGTGGCCTGGGCGGGACTGGGGAGTGGCGAGCCCTCAGATGCTGCATA**TAA**GCAGCTGCTTTTTGCCTGTACTGGGTCTCTCTGGTTAGACCAGATCTGAGCCTGGGAGCTCTCTGGCTAACTAGGGAACCCACTGCT**TAA**GCCTCAATAAAGCTTGCCTTGAGTGCTTCAAGTAGTGTGTGCCCGTCTGTTGTG**TGA**CTCTGG**TAA**CTAGAGATCCCTCAGACCCTTTTAGTCAGTGTGGAAAATCTC**TAG**CA


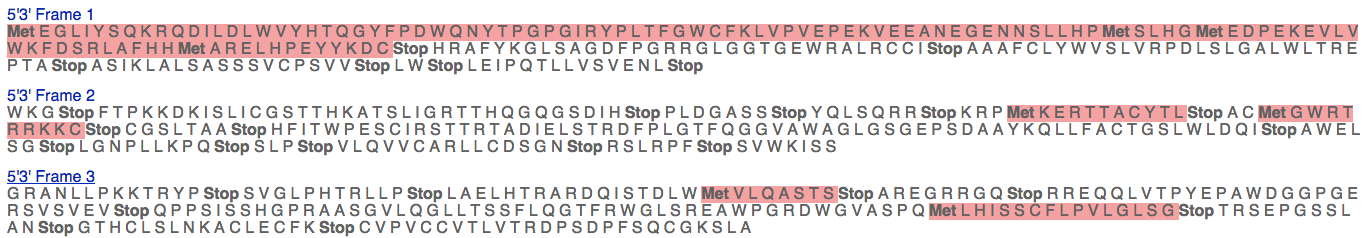


**SubB altered**

GCCGCCACCATGGAAGGGCTAATTTACTCCCAAAAAAGACAAGATATCCTTGATCTGTGGGTCTACCACACACAAGGCTACTTCCCTGATTGGCAGAACTACACACCAGGGCCAGGGATCAGATATCCACTGACCTTTGGATGGTGCTTCAAGCTAGTACCAGTTGAGCCAGAGAAGGTAGAAGAGGCCAATGAAGGAGAGAACAACAGCTTGTTACACCCT**GTG**AGCCTGCATGGG**GTG**GAGGACCCGGAGAAAGAAGTGTTAGTGTGGAAGTTTGACAGCCGCCTAGCATTTCATCAC**GTG**GCCCGAGAGCTGCATCCGGAGTACTACAAGGACTGC**GGA**CATCGAGCTTTCTACAAGGGACTTTCCGCTGGGGACTTTCCAGGGAGGCGTGGCCTGGGCGGGACTGGGGAGTGGCGAGCCCTCAGATGCTGCATA**GAA**GCAGCTGCTTTTTGCCTGTACTGGGTCTCTCTGGTTAGACCAGATCTGAGCCTGGGAGCTCTCTGGCTAACTAGGGAACCCACTGCT**GAA**GCCTCAATAAAGCTTGCCTTGAGTGCTTCAAGTAGTGTGTGCCCGTCTGTTGTG**GGA**CTCTGG**GAA**CTAGAGATCCCTCAGACCCTTTTAGTCAGTGTGGAAAATCTC**GAG**CA

**
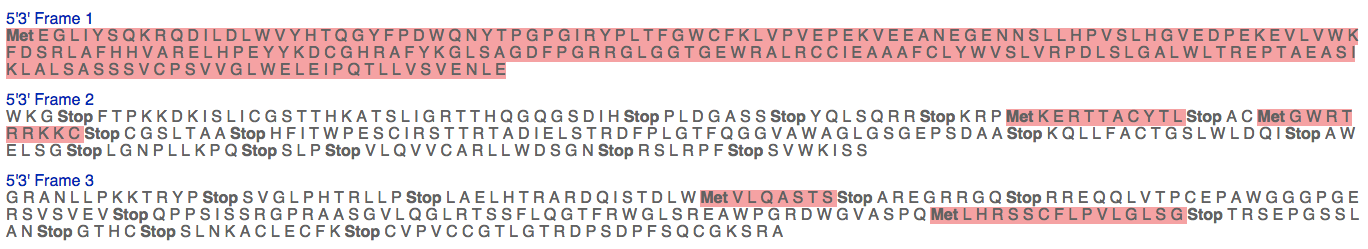
**

**Amino acid changes**

ATG to GTG, M to V

TGA to GGA, Stop to G

TAG to GAG, Stop to E

TAA to GAA, Stop to E

Underlined = internal start or stop codon

Red = added or altered nucleotides

Green = non-coding Kozak sequence

**SubC**

GCCGCCACCATGGAAGGGTTAATTTACTCTAAGAAAAGGCAAGAGATCCTTGATTTGTGGGTCTATCACACACAAGGCTACTTCCCTGATTGGCAAAACTACACACCGGGACCAGGGGTCAGATACCCACTGACCTTTGGATGGTGCTTCAAGCTAGTACCAGTTGACCCAAGGGAAGTAGAAGAGGCCAACGAAGGAGAAAACAACTGTTTGCTACACCCT**ATG**AGCCAGCATGGA**ATG**GAGGATGAACACAGAGAAGTATTAAAGTGGAAGTTTGACAGTCACCTAGCACGCAGACAC**ATG**GCCCGCGAGCTACATCCGGAGTATTACAAAGACTGC**TGA**CACAGAAGGGACTTTCCGCTGGGACTTTCCACTGGGGCGTTCCAGGAGGTGTGGTCTGGGCGGGACTGGGAGTGGTCAACCCTCAGATGCTGCATA**TAA**GCAGCTGCTTTTCGCCTGTACTGGGTCTCTCTAGGTAGACCAGATCTGAGCCTGGGAGCTCTCTGGCTATCTAGGGAACCCACTGCT**TAA**GCCTCAATAAAGCTTGCCTTGAGTGCTCTAAGTAGTGTGTGCCCTCTGTTT**TGA**CTCTGG**TAA**CTAGAGATCCCTCAGACCCTTTTGGTAGTGAGGAAATCTCTAGCA


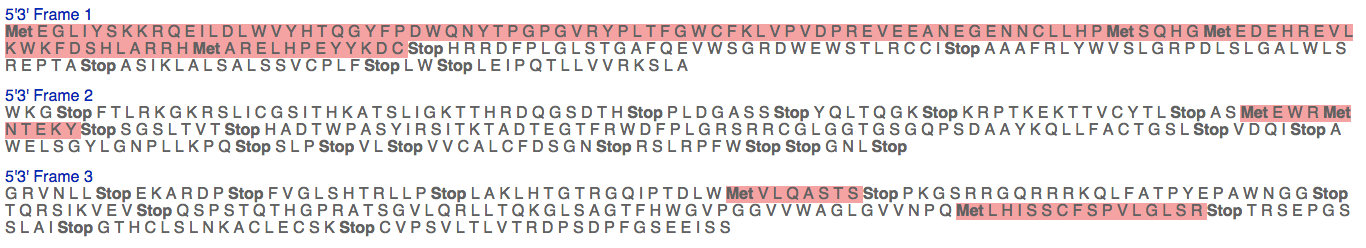


**SubC altered**

GCCGCCACCATGGAAGGGTTAATTTACTCTAAGAAAAGGCAAGAGATCCTTGATTTGTGGGTCTATCACACACAAGGCTACTTCCCTGATTGGCAAAACTACACACCGGGACCAGGGGTCAGATACCCACTGACCTTTGGATGGTGCTTCAAGCTAGTACCAGTTGACCCAAGGGAAGTAGAAGAGGCCAACGAAGGAGAAAACAACTGTTTGCTACACCCT**GTG**AGCCAGCATGGA**GTG**GAGGATGAACACAGAGAAGTATTAAAGTGGAAGTTTGACAGTCACCTAGCACGCAGACAC**GTG**GCCCGCGAGCTACATCCGGAGTATTACAAAGACTGC**GGA**CACAGAAGGGACTTTCCGCTGGGACTTTCCACTGGGGCGTTCCAGGAGGTGTGGTCTGGGCGGGACTGGGAGTGGTCAACCCTCAGATGCTGCATA**GAA**GCAGCTGCTTTTCGCCTGTACTGGGTCTCTCTAGGTAGACCAGATCTGAGCCTGGGAGCTCTCTGGCTATCTAGGGAACCCACTGCT**GAA**GCCTCAATAAAGCTTGCCTTGAGTGCTCTAAGTAGTGTGTGCCCTCTGTTT**GGA**CTCTGG**GAA**CTAGAGATCCCTCAGACCCTTTTGGTAGTGAGGAAATCTCTAGCA


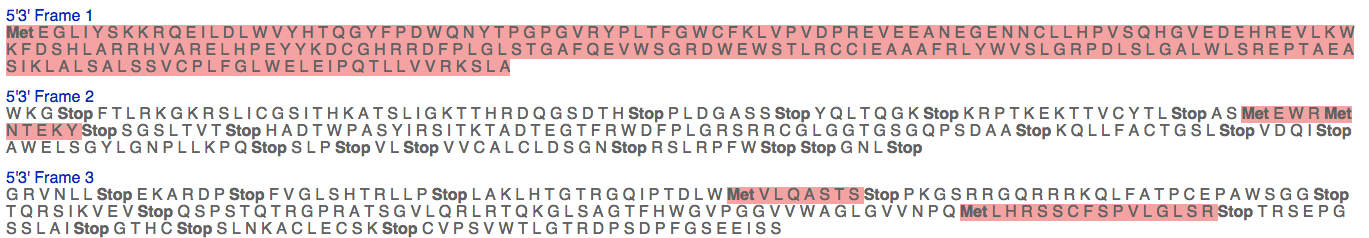


**1, 2, 18, 19 reporter**

1 & 2 (overlapping, 1bp different)

CTGCTTATATGCAGCATCTG**AGGG**

18 & 19 (overlapping, 1bp different)

**
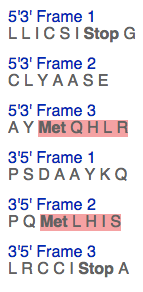
**ACAAAGACTGCTGACACAGA**AGGG**


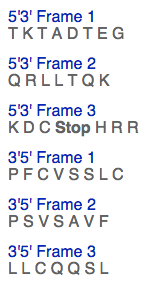


GCCGCCACCATGACAAAGACTGCTGACACAGA**AGGGCCCT**CAGATGCTGCATATAAGCAG


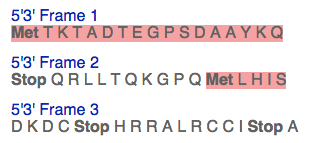


**LTR reporter cloning**

Clone gBlock into HindIII/NcoI sites of pscAAV-MND-eGFP.

Green = vector overlap

Green & underlined = remaining HindIII or NcoI restriction site nucleotide or start codon

Blue = Kozak sequence

Orange & underlined = Start codon

Black = LTR

**GroupM-gBlock (696bp)**

GAGCTCGTTTAGTGAACCGTCAGATCAGCCGCCACCATGGAAGGGTTAATTTACTCCAAGAAAAGACAAGAGATCCTTGATCTGTGGGTCTATCACACACAAGGCTACTTCCCTGATTGGCAAAACTACACACCAGGGCCAGGGATCAGATATCCACTGACCTTTGGATGGTGCTTCAAGCTAGTACCAGTTGACCCAGAGGAAGTAGAAGAGGCCAATGAAGGAGAGAACAACTGCTTGTTACACCCTGTGAGCCAGCATGGAGTGGAGGATGAAGAGAGAGAAGTGTTAGTGTGGAAGTTTGACAGCCGCCTAGCACTCAGACACGTGGCCCGAGAGCTGCATCCGGAGTACTACAAAGACTGCGGACACAGAAGTTTCTACAGGACTTTCCGCTGGGGACTTTCCAGGGGGGTGTGGTCTGGGCGGGACTGGGGAGTGGCCAACCCTCAGGTGCTGCATATAAGCAGCTGCTTTTCGCCTGTACTGGGTCTCTCTAGTGAGACCAGATCTGAGCCTGGGAGCTCTCTGGCTAGCGAGGGAACCCACTGCTTAAGCCTCAATAAAGCTTGCCTTGAGTGCTTTAAGGAGTGTGTGCCCGTCTGTTGTGTGACTCTGGTAACTAGAGATCCCTCAGACCATTTTAGTCAGTGTGGAAAATCTCTAGCAGTGAGCAAGGGCGAGGAGCTGTTCACC

**
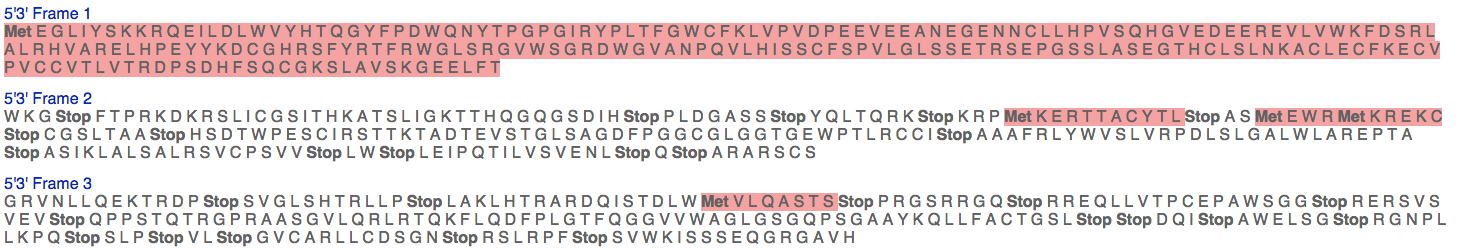
**

**SubA-gBlock (702bp)**

GAGCTCGTTTAGTGAACCGTCAGATCAGCCGCCACCATGGATGGGTTAATTTACTCCAGGAAAAGACAAGAAATCCTTGATCTGTGGGTCTACCACACACAAGGCTACTTCCCTGATTGGCAGAATTACACACCAGGGCCAGGGATCAGATACCCACTAACATTTGGATGGTGCTTCAAGCTAGTACCAGTTGATCCAGATGAAGTAGAGAAGGCTACTGAGGGAGAGAACAACAGCCTATTACACCCTATATGCCAACATGGAGTGGATGATGAGGAGAGAGAAACATTAGTGTGGAAGTTTGACAGCCGCCTGGCACTAAAACACAGAGCCCGAGAGCTGCATCCGGAGTTCTACAAAGACTGCGGACACAGAAGTTGCGGACAGGGACTTTCCGCTGGGGACTTTCCAGGGGAGGTGTGGTTTGGGCGGAGTTGGGGAGTGGCTAACCCTCAGGTGCTGCATATAAGCAGCTGCTTTTCGCCTGTACTGGGTCTCTCTTGTGAGACCAGATCGAGCCTGGGAGCTCTCTGGCTAGCTAGGGAACCCACTGCTGAAGCCTCAATAAAGCTTGCCTTGAGTGCTTCAAGTAGTGTGTGCCCGTCTGTTGTGGGACTCTGGGAACTAGAGATCCCTCAGACCACTCTAGACTGTGTAAAAATCTCTAGCAGTGGCGTGAGCAAGGGCGAGGAGCTGTTCACC

**
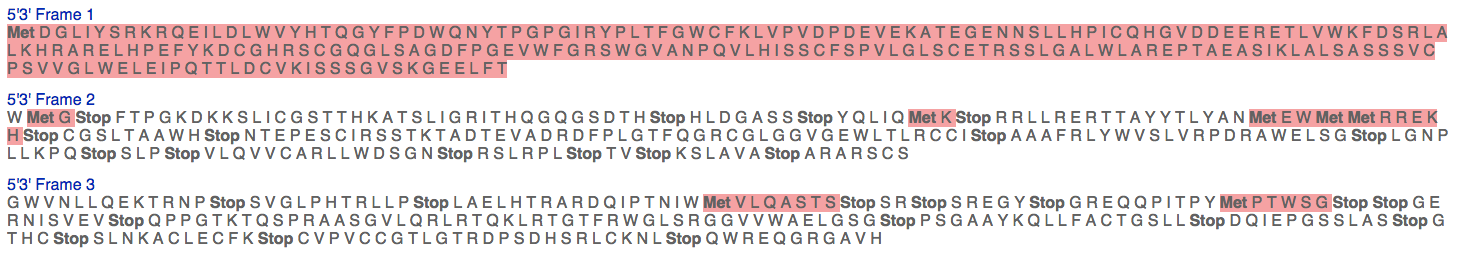
**

**SubB-gBlock (699bp)**

GAGCTCGTTTAGTGAACCGTCAGATCAGCCGCCACCATGGAAGGGCTAATTTACTCCCAAAAAAGACAAGATATCCTTGATCTGTGGGTCTACCACACACAAGGCTACTTCCCTGATTGGCAGAACTACACACCAGGGCCAGGGATCAGATATCCACTGACCTTTGGATGGTGCTTCAAGCTAGTACCAGTTGAGCCAGAGAAGGTAGAAGAGGCCAATGAAGGAGAGAACAACAGCTTGTTACACCCTGTGAGCCTGCATGGGGTGGAGGACCCGGAGAAAGAAGTGTTAGTGTGGAAGTTTGACAGCCGCCTAGCATTTCATCACGTGGCCCGAGAGCTGCATCCGGAGTACTACAAGGACTGCGGACATCGAGCTTTCTACAAGGGACTTTCCGCTGGGGACTTTCCAGGGAGGCGTGGCCTGGGCGGGACTGGGGAGTGGCGAGCCCTCAGATGCTGCATAGAAGCAGCTGCTTTTTGCCTGTACTGGGTCTCTCTGGTTAGACCAGATCTGAGCCTGGGAGCTCTCTGGCTAACTAGGGAACCCACTGCTGAAGCCTCAATAAAGCTTGCCTTGAGTGCTTCAAGTAGTGTGTGCCCGTCTGTTGTGGGACTCTGGGAACTAGAGATCCCTCAGACCCTTTTAGTCAGTGTGGAAAATCTCGAGCAAGTGAGCAAGGGCGAGGAGCTGTTCACC

**
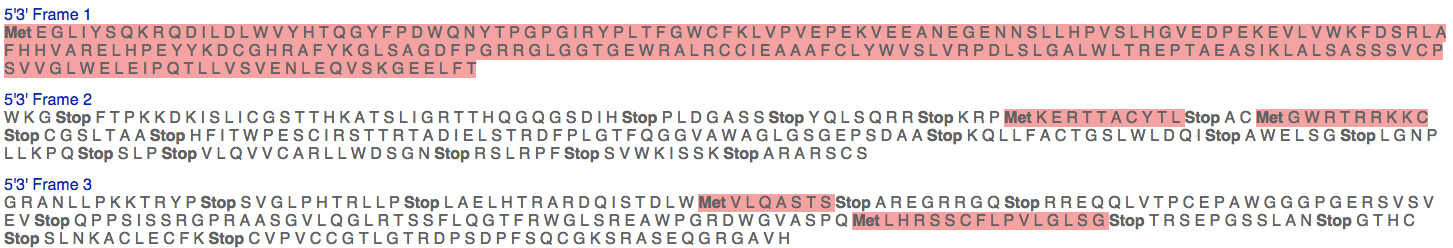
**

**SubC-gBlock (693bp)**

GAGCTCGTTTAGTGAACCGTCAGATCAGCCGCCACCATGGAAGGGTTAATTTACTCTAAGAAAAGGCAAGAGATCCTTGATTTGTGGGTCTATCACACACAAGGCTACTTCCCTGATTGGCAAAACTACACACCGGGACCAGGGGTCAGATACCCACTGACCTTTGGATGGTGCTTCAAGCTAGTACCAGTTGACCCAAGGGAAGTAGAAGAGGCCAACGAAGGAGAAAACAACTGTTTGCTACACCCTGTGAGCCAGCATGGAGTGGAGGATGAACACAGAGAAGTATTAAAGTGGAAGTTTGACAGTCACCTAGCACGCAGACACGTGGCCCGCGAGCTACATCCGGAGTATTACAAAGACTGCGGACACAGAAGGGACTTTCCGCTGGGACTTTCCACTGGGGCGTTCCAGGAGGTGTGGTCTGGGCGGGACTGGGAGTGGTCAACCCTCAGATGCTGCATAGAAGCAGCTGCTTTTCGCCTGTACTGGGTCTCTCTAGGTAGACCAGATCTGAGCCTGGGAGCTCTCTGGCTATCTAGGGAACCCACTGCTGAAGCCTCAATAAAGCTTGCCTTGAGTGCTCTAAGTAGTGTGTGCCCTCTGTTTGGACTCTGGGAACTAGAGATCCCTCAGACCCTTTTGGTAGTGAGGAAATCTCTAGCAGTGAGCAAGGGCGAGGAGCTGTTCACC

**
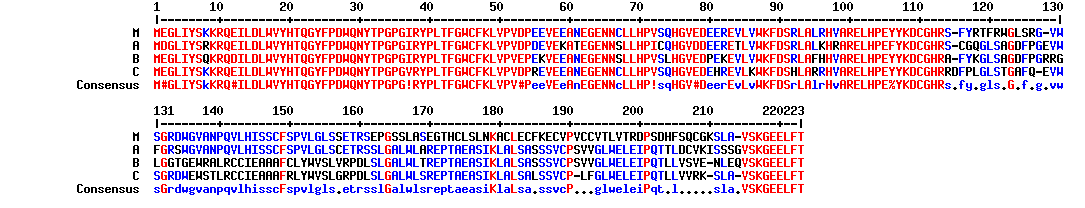
**

LTR reporter amino acid alignment (VSKGEELFT = eGFP)

**1-2-18-19 reporter cloning**

Clone gBlock into HindIII/NcoI sites of pscAAV-MND-eGFP.

Green = vector overlap

Green & underlined = remaining HindIII or NcoI restriction site nucleotide or start codon

Blue = Kozak sequence

Orange & underlined = Start codon

Purple & black Underlined = saCas9 sgRNA target sites

**1-2-18-19-gBlock (114bp)**

GAGCTCGTTTAGTGAACCGTCAGATCAGCCGCCACCATGACAAAGACTGCTGACACAGA**AGGGCCCT**CAGATGCTGCATATAAGCAGGTGAGCAAGGGCGAGGAGCTGTTCACC


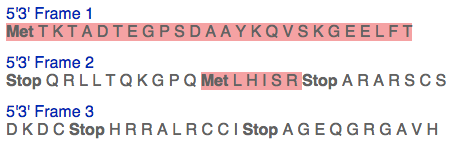

Supplement: Supplementary file 5 — Supplementary methods: c reporter design. (DOCX 1641 kb) [file 12915_2018_544_MOESM5_ESM.docx]
